# The tendency to recreate ancestral CG dinucleotides in the human genome

**DOI:** 10.1186/1471-2148-11-3

**Published:** 2011-01-05

**Authors:** Mingkun Li, Su-Shing Chen

**Affiliations:** 1 CAS-MPG Partner Institute of Computational Biology, Shanghai Institutes of Biological Sciences, Chinese Academy of Sciences, 200000, Shanghai, PR China; 2 Department of Evolutionary Genetics, Max Planck Institute for Evolutionary Anthropology, D04103, Leipzig, Germany

## Abstract

**Background:**

The CG dinucleotides are known to be deficient in the human genome, due to a high mutation rate from 5-methylated CG to TG and its complementary pair CA. Meanwhile, many cellular functions rely on these CG dinucleotides, such as gene expression controlled by cytosine methylation status. Thus, CG dinucleotides that provide essential functional substrates should be retained in genomes. How these two conflicting processes regarding the fate of CG dinucleotides - i.e., high mutation rate destroying CG dinucleotides, vs. functional processes that require their preservation remains an unsolved question.

**Results:**

By analyzing the mutation and frequency spectrum of newly derived alleles in the human genome, a tendency towards generating more CGs was observed, which was mainly contributed by an excess number of mutations from CA/TG to CG. Simultaneously, we found a fixation preference for CGs derived from TG/CA rather than CGs generated by other dinucleotides. These tendencies were observed both in intergenic and genic regions. An analysis of Integrated Extended Haplotype Homozygosity provided no evidence of selection for newly derived CGs.

**Conclusions:**

Ancestral CG dinucleotides that were subsequently lost by mutation tend to be recreated in the human genome, as indicated by a biased mutation and fixation pattern favoring new CGs that derived from TG/CA.

## Background

DNA methylation is central to important biological processes, including X chromosome inactivation[1], genetic imprinting [2], gene-expression regulation [3-5], and the defense mechanisms against parasitic DNA and transposons [6,7]. DNA methylation also plays an important role in pathological processes such as cancer [8], is closely related with histone modification and RNA-associated silencing [9], and may be involved in genomic instability [10].

DNA methylation in mammals is carried out by three methyltransferases (DNMT1, DNMT3A, DNMT3B)[11] that target the cytosine in CG dinucleotides. A stochastic model for the maintenance of methylation during DNA replication has been proposed that allows accurate epigenetic inheritance[9,11]. However, CG dinucleotides also experience a high mutation rate from cytosine to thymine through the deamination of 5-methylcytosine and a less-than-perfect repair mechanism for resulting G/T mismatches [12-14], which calls into question the stability of methylation of CG dinucleotides as an important epigenetic marker.

In the human genome, the number of CG dinucleotides is ~25% of that expected given observed base frequencies, which is due to a high mutation rate from 5-methylated CG to TG and its complementary pair CA[15]. 15% of all CGs are clustered in so called "CpG islands", which are often located at the 5' end of genes and overlap with the promoter region, especially in housekeeping genes [16]. Their methylation status is highly associated with gene expression [3], but despite this apparent functional constraint, loss of CpG islands has been observed as a common event in mammalian genomes [15,17,18].

Moreover, multiple pathways exist that preserve CG dinucleotides in the genome, including a lack of methylation of CGs in CpG islands and a strong correction bias towards C/G following T/G mismatches [14,19,20]. Two enzymes (TDG and MBD4) have been found to selectively remove the thymine from a T:G mismatch in the context of CpG dinucleotides [21,22].

In our previous study [23], we observed a strong fixation bias in favor of derived C/G alleles at CG-related sites (dinucleotides that differ by one-step mutation from CG: CT, CA, CC, AG, TG, GG) compared with C/G alleles at CG-free sites (positions not preceded by C or followed by G). TG and CA, which are predominant at CG-related sites, were suspected to contribute this bias. Since a large fraction of TG/CA sites are originally derived from deamination of of methylated CGs [24], we hypothesize that recreating ancestral CGs may be the driving force behind this bias. Data released by the HapMap project and various genome projects afford us the opportunity to test this hypothesis.

By analyzing the mutation and frequency spectrum of different derived alleles, while taking the sequence context into account, a significant mutation and fixation preference towards transition-generated CGs (CG derived from CA or TG) was observed, while no such preference was observed for transversion-generated CGs (CG derived from CC, GG, CT, AG). This mutation and fixation preference enables the recreation of ancestral CG dinucleotides lost via hypermutation to CA/TG.

## Methods

### Data collection

Human SNP data were retrieved from dbSNP (ftp://ftp.ncbi.nih.gov/snp/, Build 124), and curated by the method described elsewhere[25]. Briefly, the following SNPs were selected: nonindel, biallelic, uniquely mapped to nonrepetitive sequences, validated, and with at least 100 nucleotides (nt) of flanking sequence available both upstream and downstream from the SNP.

Sequences and reads for multiple species were retrieved from the NCBI Nucleotide Databasehttp://www.ncbi.nih.gov/sites/entrez?db=nuccore and Traces Databasehttp://www.ncbi.nih.gov/Traces. Finally, the chimpanzee(Pan troglodytes, whole genome, 2.82G), gorilla(Gorilla, 9.54G, unassembled), orangutan (Pongo, 18.37G, unassembled), and Gibbon(Nomascus, 18.64G, unassembled) genome sequences were used as outgroups to infer the ancestral allele for the human SNP.

The allele frequencies at each SNP site were estimated from flat files downloaded from the International HapMap Project (http://hapmap.ncbi.nlm.nih.gov/downloads/frequencies/, Build 23). The derived-allele frequency (DAF) for each SNP was estimated in three populations (unrelated individuals only): Yoruba(YRI), Utah residents with ancestry from northern and western Europe(CEU), Japanese and Chinese(ASN); only those SNPs successfully genotyped in all chromosomes (120, 120, 180 respectively) were kept for further analysis.

### Inferring ancestral states of the SNPs

Inferring ancestral states using parsimony may be unreliable if the mutation or substitution rate is high[26]. In mammalian genomes, CG dinucleotides have a very high mutation rate [27]; therefore, two methods were applied to ensure accurate inference of the ancestral state.

First, MegaBLAST was used to map the human SNPs and their flanking sequences(100 bp on each side) to the four outgroup sequences (chimpanzee, gorilla, orangutan, gibbon)[28]. We inferred the ancestral allele only when the chimpanzee, gorilla, orangutan and gibbon alleles were identical and matched one of the human SNP alleles. A PERL script was written to retrieve the human SNPs and their corresponding outgroup alleles from the MegaBLAST result.

Second, we also downloaded a dataset of 14.3 Mb of well curated human/chimpanzee/baboon DNA alignments (http://pbil.univ-lyon1.fr/datasets/MeunierDuret2004/data)[29] and the corresponding human/rhesus sequences from http://genome.ucsc.edu. Blast was used to construct new alignments, and the final alignment length was shortened to 9.4 Mb. Human SNPs that map to the aligned chimp/baboon/rhesus sequences were retrieved and the ancestral state inferred as described above. Given the branch lengths estimated by Steiper and Young [30] for the primate phylogeny, reliable inference of the ancestral state can be obtained using these three outgroups even at CG related positions (Additional file 1).

For CpG islands, a single outgroup (chimpanzee) was used to infer the mutation direction to maximize the sample size, and the frequency spectrum was then corrected by Hernadez et al.'s method (Additional file 2) [26].

### Identification of SNPs located in CpG islands

We identified 37,503 CpG islands in the human genome using the CGi130 program developed by Takai and Jones (http://cpgislands.usc.edu/)[31,32]. The following search criteria, considered stringent according to the authors, were used: i) GC content ≥ 55%, ii) ObsCG/ExpCG ≥ 0.65, and iii) length ≥ 500 bp.

### Gene annotation

The gene annotation for each SNP in our study was retrieved from the ENSEMBL database (ftp://ftp.ensembl.org/pub/, version 45), and SNPs were classified as intergenic, intronic, coding, 5'UTR, 3'UTR, 5'upstream, or 3'downstream. Any SNP classified into more than one category was removed from the analysis. This left us with 915243 intergenic, 634809 intronic, 18333 coding, 2055 5'UTR, 11329 3'UTR, 38398 5'upstream and 40749 3'downstream SNPs.

### Detection of positive selection

A Long-Rang Haplotype test was utilized to detect recent positive selection. Specifically, the Integrated Haplotype Score (iHS) was calculated for each SNP, as described elsewhere[33]. We used the suggested cut-off value, namely an extreme positive (iHS > 2) or negative (iHS < -2) iHS score (indicating a longer haplotype associated with the ancestral or the derived allele, respectively), as an indication of recent positive selection.

### Recombination hotspots and coldspots

The locations of recombination hotspots and coldspots were obtained from a fine-scale genetic map estimated from patterns of genetic variation, which provides a kilobase-scale resolution of recombination rates [34,35].

## Results

### Mutation and fixation bias towards recreating ancestral CGs

Following our previous observation of a strong fixation bias in favor of derived C/G alleles at CG-related sites[23], we re-examined the mutation and fixation pattern of CG dinucleotides on a genome-wide scale, while taking the sequence context into consideration. The CGs generated by a single mutation from other dinucleotides were classified into transition-generated CGs (Tsg, CG derived from CA/TG) and transversion-generated CGs (Tvg, CG derived from CC/CT/AG/GG). Correspondingly, the dinucleotides derived by mutations from CG are divided into transition-damaged CGs (Tsd, CA/TG derived from CG) and transversion-damaged CGs (Tvd, CC/CT/AG/GG derived from CG). For comparison, the same mutations located in other sequence contexts (not preceded by C or followed by G) were used as the control data for each of these mutation types. Analyses were also done separately for CpG islands and non-CpG islands, since they have very different mutation patterns and nucleotide composition.

In the non-CpG island region, the number of mutations in each annotation category varies substantially, ranging from 819 for transversion-damaged CGs to 19631 for transition-generated CGs for YRI population (Table 1; Additional file 3 for CEU and Additional file 4 for ASN). A total of 9293 mutations are from CG to other dinucleotides, among which 91% are to CA/TG, which is significantly larger than the fraction of transitions for the control data (62%, P < 2.2e-16, Fisher Exact test). There are 26391 mutations generating new CGs from other dinucleotides, of which 74% are transitions (derived from CA/TG), which is significantly larger than the fraction of transitions at control positions (64%, P < 2.2e-16, Fisher Exact test). Such a biased process, dominated by mutations between CGs and CAs/TGs, predicts an increase in CG dinucleotides in the non-CpG island region, and differs significantly from the trend demonstrated by the control data (67831:64859, P < 2.2e-16, Fisher Exact test).

**Table 1:** Number of SNPs and average derived-allele frequency (DAF) for different mutation types in different annotation categories

	Non-CpG island region				CpG island region			
	
	Intergenic		Genic		Intergenic		Genic	
	
Mutation type*	SNPs	DAF	SNPs	DAF	SNPs	DAF	SNPs	DAF
All	88447	0.324	79241	0.317	1053	0.34	3120	0.316
Tsd	3752	0.314	4722	0.303	158	0.301	461	0.3
C-Tsd	22539	0.311	19525	0.303	286	0.324	861	0.309
Tsg	10235	0.353	9396	0.344	100	0.406	240	0.373
C-Tsg	21567	0.33	19866	0.326	138	0.401	403	0.339
Tvd	419	0.333	400	0.322	66	0.312	196	0.325
C-Tvd	14100	0.317	11667	0.309	140	0.337	461	0.3
Tvg	3415	0.306	3345	0.304	50	0.385	169	0.309
C-Tvg	12481	0.33	10935	0.319	108	0.346	354	0.302

* The symbols used here are identical to that in the text, with the prefix of C- denoting the corresponding control dataset.

In the CpG island region, a different pattern was found: 559 CGs are generated by mutations from other dinucleotides while 881 CGs are destroyed by mutations; there is thus a trend toward decreasing the number of CGs. The trend is in accordance with that shown by the control data for CpG island regions (1003 Cs/Gs generated vs. 1748 Cs/Gs destroyed, P = 0.139, Fisher Exact test), but differs significantly from that in the non-CpG island region (26391 CGs generated vs. 9293 CGs destroyed, P < 2.2e-16, Fisher Exact test).

**Figure 1:**
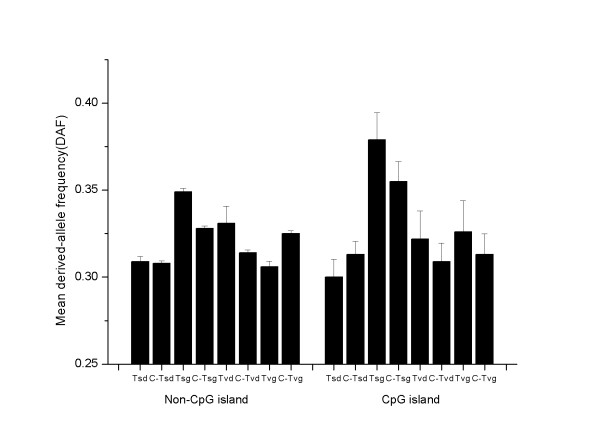
**Derived-allele frequency for different mutation types in YRI**. Left histogram is for non-CpG island regions, right histogram is for CpG island regions. Abbreviations are the same as in the text, with the prefix of C- denoting the corresponding control dataset.

Derived-allele frequencies (DAF) are shown in Figure 1 for YRI (Additional file 5 for CEU, Additional file 6 for ASN). In the non-CpG island regions, when the derived allele was not preceded by C or followed by G, transition-generated CGs (Tsg) and transversion-generated CGs (Tvg) had a similar average DAF in the control data (0.328 vs. 0.325, P = 0.1019, Mann-Whitney U test), indicating no intrinsic DAF difference for these two types of mutations (A/T- > G/C and C/G/T/A- > G/C/G/C). However, the average DAF of transition-generated CGs (Tsg) was significantly greater than for the control data (0.349 vs. 0.328, P = 7.6e-12, Mann-Whitney U test), whereas transversion-generated CGs (Tvg) had a significantly lower average DAF than for the control data (0.325 vs. 0.306, P = 1.3e-8, Mann-Whitney U test). Overall, it suggests a higher probability of fixation for transition-generated CGs (Tsg) than for transversion-generated CGs (Tvg)(P < 2.2e-16, Mann-Whitney U test). Meanwhile, the average DAF of CGs destroyed by transitions (i.e., CAs/TGs derived from CGs) did not differ significantly from the control data (0.309 vs. 0.308, P = 0.65, Mann-Whitney U test), nor was the average DAF of CGs destroyed by transversions (i.e., CC/GG/CT/AG derived from CGs) significantly different from the control data (0.331 vs 0.314, P = 0.65, Mann-Whitney U test).

For CpG islands, the DAF for YRI is shown in Figure 1, and in Additional file 5 for CEU and Additional file 6 for ASN. Transition-generated CGs (Tsg) had a much higher DAF than transversion-generated CGs (Tvg)(0.379 vs. 0.326 for YRI, P = 0.023, Mann-Whitney U test). However, the control data showed the same trend (0.355 vs. 0.313, P = 0.0058, Mann-Whitney U test).

To investigate the influence of potential errors in assigning ancestral alleles on the above analyses, 9.4 Mb multiple sequence alignments of human/chimpanzee/baboon/rhesus were created by another method and analyzed. The same mutation and fixation patterns were observed (Additional file 7, Additional file 8).

### Selection on the CG dinucleotides?

To investigate if the above results might reflect selection on functional elements, the SNPs were classfied into four categories: intergenic non-CpG island; genic non-CpG island; intergenic CpG island; and genic CpG island (genic regions included the sites annotated as intronic, coding, 5'UTR, 3'UTR, 5'upstream, 3'downstream). Normally, intergenic, non-CpG island regions are regarded as neutral, so if selection is playing a role in the trends we observed, there should be differences between genic and intergenic patterns. However, we found similar patterns in genic and intergenic regions (Table 1).

We also applied the iHs test for signals of recent positive selection on newly-generated CG dinucleotides. Newly-generated CGs with iHs < -2 may be under positive selection while whose iHs > 2 may be under negative selection. Among 16843 transition-generated CGs, there were 322 (1.9%) with iHs < -2, which is not significantly different from the fraction of transition-generated Cs/Gs in the control data with iHs < -2 (2.1%, P = 0.1462, Fisher's Exact test). Similar results were obtained for transversion-generated CGs (Table 2). Overall, there is no evidence that newly-generated CGs are under recent positive selection.

**Table 2:** Recent selection on the newly generated CG dinucleotides in different mutation categories

	Transition-generated CG			Control for transition-generated CG			Transversion-generated CG			Control for transversion-generated CG		
	
Population	**Proportion indicating positive selection**^**1.**^	**DAF of the positive selected CG**^**2.**^	**general DAF**^**3.**^	Proportion indicating positive selection	DAF of the selected CG	general DAF	Proportion indicating **negative selection**^**1.**^	**DAF of the negative selected CG**^**2.**^	general DAF	Proportion indicating negative selection	DAF of the selected CG	general DAF
YRI	0.019	0.354	0.370	0.021	0.348	0.353	0.021	0.319	0.333	0.021	0.338	0.349
CEU	0.015	0.425	0.412	0.016	0.446	0.403	0.022	0.375	0.387	0.026	0.353	0.399
ASN	0.014	0.465	0.428	0.016	0.452	0.418	0.024	0.345	0.407	0.026	0.368	0.414

^1. ^Proportion of the SNPs whose iHs < -2 (positive selection) or iHs > 2 (negative selection) among all SNPs;

^2. ^DAF for SNPs with iHs < -2(positive selection) or iHs > 2(negative selection);

^3. ^DAF for all SNPs.

With the phylogeny of ((((((CG,CA)CA)CA)CA)CG)CG) for ((((((human1,human2)chimpanzee)gorilla)orangutan)gibbon)rhesus)(phylogeny tree is shown in Additional file 9), phylogenetic analyses can reveal situations where an ancestral CG mutated to CA and then back to CG in the human lineage. A total of 34 SNPs were identified as back-mutations to CGs, of which three had a significant iHs value (Table 3), which is higher than the fraction of mutations with significant iHs values in the control data (P = 0.011, Chi-square test). All three significant iHs values involve transition-generated CGs rather than transversion-generated CGs.

**Table 3:** Recent selection on the backmutated CG dinucleotides

			YRI		CEU		ASN	
		
SNP ID	Mutation type	Position	iHs	DAF	iHs	DAF	iHs	DAF
rs9409314	ATG->ACG	intergenic	2.1	0.8	2.39	0.339	2.56	0.545
rs7977620	CAT- > CGT	NAV3 upstream	1.14	0.921	2.37	0.558	2.14	0.6
rs4687991	CAT- > CGT	intergenic	-0.97	0.788	2.15	0.458	1.47	0.649

### Fixation bias in the recombination hotspots and coldspots

**Figure 2:**
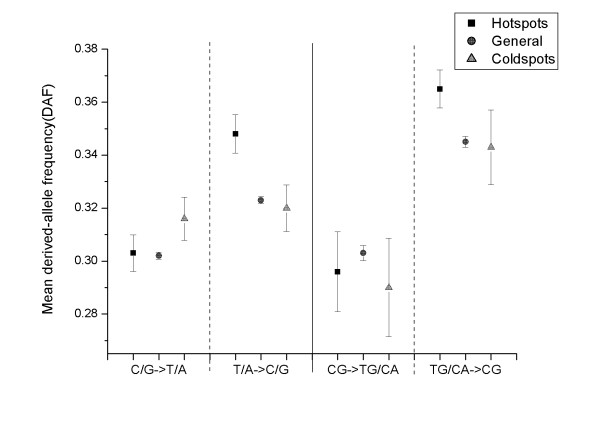
**Derived-allele frequency for different mutation types in recombination hotspots and coldspots in YRI**. Columns on the left of the solid line represent the pattern without the influence of the CG context (mutations not preceded by C or followed by G); columns on the right of the solid line represent the pattern with CG context.

BGC (Biased Gene Conversion) is a phenomenon universal in vertebrate genomes and results in fixation bias towards the C/G allele in the process of repair of double-strand breaks induced by recombination[36]. If the BGC effect is responsible for the fixation preference observed here (namely, transition-generated CGs are more prone to be fixed in the genome compared with the control data), then recombination hotspots and coldspots should differ. The result is shown in Figure 2 for YRI (Additional file 10 for CEU, Additional file 11 for ASN).

In the recombination hotspots, C/G derived from T/A had a much higher DAF than T/A that derived from C/G (0.347 vs. 0.304, P < 2.2e-16, Mann-Whitney U Test), which is in accordance with BGC effect. Meanwhile, the DAF was significantly greater for CG derived from CA/TG than for the control data (0.365 vs. 0.348, P < 2.2e-16, Mann-Whitney U Test), whereas the DAF of TG/CA derived from CG didn't differ significantly from the control data (0.296 vs. 0.304, P = 0.546, Mann-Whitney U Test). In the recombination coldspots, T/A derived from C/G had the same average DAF as that of C/G derived from T/A (0.316 vs. 0.320, P = 0.989, Mann-Whitney U Test), indicating that the fixation bias toward C/G disappeared in regions of low recombination, which is expected under BGC model. However, the DAF of CG derived from CA/TG is still higher than that of CA/TG derived from CG (0.343 vs. 0.290, P = 0.1717, Mann-Whitney U test), as well as that of the control data (0.343 vs. 0.320, P = 0.230, Mann-Whitney U test), but not statistically significant. Overall, these results indicate that the tendency observed in our study might be partially explained by recombination, further study is needed to address this question.

## Discussion

### Mutation and fixation bias recreating ancestral CGs

The observation that CG dinucleotides are under-represented in vertebrate genomes has received much attention. Much evidence suggests that the observed deficiency of CG dinucleotides is caused by hypermutable methylated cytosine, which mainly exists as the CG dinucleotide in vertebrate genomes[24]. This is further supported by the observation that 90% of the mutations from CGs are transitions to CA/TG in our study, accompanied with the overrepresentation of TG/CA in the genome reported by others [24]. More directly, when the CGs in CpG islands become methylated, CGs mutate to TG/CA at a comparatively high rate [37]. It is estimated that the advent of heavily methylated genomes dates to approximately 450 million years ago, leading to a lower CG frequency [38,39]. Meanwhile, CpG islands which were thought to lack methylation were also being lost [15,17,18].

Yet the methylated CG dinucleotide acts as an essential substrate for many regulatory reactions [8]. This was believed to be a high fidelity process, until studies revealed the possibility of deleterious mutations induced by deamination of 5-methylcytosine [12-14]. The question then arose as to whether CG dinucleotides would vanish from the genome.

In our study, by analyzing the mutations involving CG dinucleotides in non-CpG island regions, a strong tendency toward increasing CG dinucleotides was observed, due to biased mutation and fixation patterns. Specifically, there are about three times as many mutations generating new CGs as mutations eliminating CGs, mainly due to the overwhelming number of mutations from CA/TG to CG. Furthermore, transition-generated CGs had a higher average DAF than transversion-generated CGs, and in comparison to control data, transition-generated CGs had a significantly higher DAF while transversion-generated CGs had a significantly lower DAF. Since most CA/TG dinucleotides are derived from deamination of 5-methylcytosine, many mutations from CA/TG to CG should be back-mutations after CG mutated to CA/TG. This scenario thereby recreates ancestral CGs in the genome while preventing novel CGs that derived from CC/CT/AG/GG.

By contrast, in CpG island regions, which are relatively abundant in CG dinucleotides, there seems to be a tendency toward reducing the number of CG dinucleotides. This may reflect evolution from a non-equilibrium GC content in such regions, as the equilibrium GC content in the human genome is estimated to be 33%-42%[29], which is much lower than the present GC content (> 55%).

### Natural selection or neutral mechanism?

Although the preference for transition-generated CGs was observed both in intergenic and genic regions, it could be driven by natural selection as many regulatory elements are located in intergenic regions [40-43]. The assumption of the selection test we applied is that, if the higher average DAF was caused by positive selection on these transition-generated CGs, a larger proportion of the CGs should exhibit signals of positive selection, compared with the control dataset. However, no difference between the CG and control data was observed, indicating that newly-generated CGs were not excessively subject to recent positive selection (Table 2). The analysis of inferred back-mutations similarly indicated that such back-mutations also were not significantly influenced by recent positive selection.

It should be noted that the failure to detect the signal of recent positive selection could also have other explanations. The present methods for detecting the signal of recent positive selection are mainly targeted at a region rather than a specific locus [33,44], and identifying causal variants is quite challenging. Since positive signals may come from hitchhiking with the selected allele, applying such methods to isolated sites rather than a genomic region may lack sufficient power. Moreover, weak selection would be difficult to detect as well. Therefore, these results do not rule out some role for selection in producing the patterns observed in this study.

### Extension of BGC model?

As an alternative to selection, the BGC model is a neutral process that increases the probability of fixation of mutations that increase GC content in the human genome [20,45]. It is therefore of interest to investigate if our findings can be explained via an extension of the BGC model in the context of CG dinucleotides, in particular the fixation preference for recreating ancestral CGs, or if some other mechanism must be invoked.

Recombination was reported to be the cause of BGC [29,46] and this is supported in our study, as the fixation preference for C/G rather than A/T exists in recombination hotspots, but not in the recombination coldspots. The fixation preference for transition-generated CGs we observed in our study might be partially driven by the recombination, as there were no significant difference between recombination hotspots and coldspots. However, more evidence is needed to address this question, as we have a rather small sample size in this analysis.

## Conclusion

In this study, we identified a trend that could be responsible for generating new CG dinucleotides in non-CpG islands, which are otherwise deficient in the human genome; namely, an excess number of mutations from CA/TG to CG, which in turn recreates ancestral CG dinucleotides in the human genome that had been previously lost by mutation. It appears that neutral process, which might be partly associated with recombination, is responsible for this trend. By contrast, in CpG island regions, which are relatively abundant in CG dinucleotides, a tendency toward reducing the number of CG dinucleotides was observed. Overall, our results shed further light on the fate of CG dinucleotides in the genome.

## Authors' contributions

LMK designed the study. LMK collected the data. LMK analyzed data. LMK and CS wrote the manuscript. All authors read and approved the final manuscript.

## Supplementary Material

Additional file 1 **Illustration of the reliability of the parsimony method to count the mutations at CpG related sites using three outgroups: chimpanzee/baboon/rhesus**. The phylogeny of the four species used to infer mutations is shown in (a). Branch length indicates the rate of substitution per site at non-CpG positions. Substitution rates at CpG sites are 10 times higher than that at non-CpG sites, (b) scenario when the parsimony method is reliable, (c) scenario when the parsimony method is not reliable. Scenario (b) is 10 times more likely to be observed than scenario (c). Comparably, if chimpanzee, baboon, or rhesus is used as a single outgroup respectively, the probability ratio between (b) and (c) would be 2:1, 0.4:1, or 0.4:1. Click here for file

Additional file 2 **Derived-allele frequency estimation in CpG island regions**. Click here for file

Additional file 3 **Number of SNPs and derived-allele frequency for different mutation types in different annotation categories in CEU**. Click here for file

Additional file 4 **Number of SNPs and derived-allele frequency for different mutation types in different annotation categories in ASN**. Click here for file

Additional file 5 **Derived-allele frequency for different mutation types in CEU**. Click here for file

Additional file 6 **Derived-allele frequency for different mutation types in ASN**. Click here for file

Additional file 7 **Number of SNPs and derived-allele frequency for different mutation types in the 9.4 Mb non-genic region**. Click here for file

Additional file 8 **Derived-allele frequency estimated by different methods of inferring the mutation direction in the 9.4 Mb DNA alignment**. Diamond symbol denotes the mean DAFs (± s.e.m). Click here for file

Additional file 9 **Phylogenetic tree for reliable back-mutations from CA to CG**. Click here for file

Additional file 10 **Derived-allele frequency for different mutation types in recombination hotspots and coldspots in CEU**. Click here for file

Additional file 11 **Derived-allele frequency for different mutation types in recombination hotspots and coldspots in ASN**. Click here for file
