# Supplementary material for: The tendency to recreate ancestral CG dinucleotides in the human genome
Source: BMC Evol Biol. 2011 Jan 5;11:3. doi: 10.1186/1471-2148-11-3 (PMC3025853; doi:10.1186/1471-2148-11-3)
Supplement: Additional file 1 — Illustration of the reliability of the parsimony method to count the mutations at CpG related sites using three outgroups: chimpanzee/baboon/rhesus. The phylogeny of the four species used to infer mutations is shown in (a). Branch length indicates the rate of substitution per site at non-CpG positions. Substitution rates at CpG sites are 10 times higher than that at non-CpG sites, (b) scenario when the parsimony method is reliable, (c) scenario when the parsimony method is not reliable. Scenario (b) is 10 times more likely to be observed than scenario (c). Comparably, if chimpanzee, baboon, or rhesus is used as a single outgroup respectively, the probability ratio between (b) and (c) would be 2:1, 0.4:1, or 0.4:1. [file 1471-2148-11-3-S1.DOC]

Human1

Human2

Chimp

Baboon

Rhesus

0.02

0.02

0.005

0.0005

0.005

TG

CG

TG

TG

TG

TG

T→C

CG

CG

TG

TG

TG

TG

C→T

C→T

C→T

P_1_=5×10^-4^

P_2_=5×10^-5^

(a)

(b)

(c)
