# Supplementary material for: The tendency to recreate ancestral CG dinucleotides in the human genome
Source: BMC Evol Biol. 2011 Jan 5;11:3. doi: 10.1186/1471-2148-11-3 (PMC3025853; doi:10.1186/1471-2148-11-3)
Supplement: Additional file 2 — Derived-allele frequency estimation in CpG island regions. [file 1471-2148-11-3-S2.DOC]

**Derived-allele frequency (DAF) estimation in CpG island regions**

In CpG island region, the sample size (SNP number) became too small when using multiple outgroups to infer the mutation direction, therefore, Hernandez et al.’s method, which could make an accurate DAF estimation using one outgroup (chimpanzee), was used[1].

However, the mutation matrix in their method can’t be used directly in the CpG island[2], because the mutation rate from CG to CA/TG is much lower in CpG island than outside. Thus, an adaptation was made to the matrix. CG dinucleotides are present with the frequency of at least 65% of its expected frequency in CpG island, while the frequency in the non-CpG island is 20% [3, 4]. Here, we use to denote the CG dinucleotdes’ equilibrium level, thus in the non-CpG island : in the CpG island ≈ 65:20.

Let u represent the rate of mutations generating CG, and v denotes the rate of mutations damaging the CG through one mutational step, then,

Only one parameter differs between that in the CpG island and non-CpG island. Therefore, in the CpG island could be estimated from other parameters. Then, the adapted mutation matrix could be used to correct the frequency in the CpG island.

1. Hernandez RD, Williamson SH, Bustamante CD: **Context dependence, ancestral misidentification, and spurious signatures of natural selection**. *Mol Biol Evol* 2007, **24**(8):1792-1800.

2. Hwang DG, Green P: **Bayesian Markov chain Monte Carlo sequence analysis reveals varying neutral substitution patterns in mammalian evolution**. *Proc Natl Acad Sci U S A* 2004, **101**(39):13994-14001.

3. Sved J, Bird A: **The expected equilibrium of the CpG dinucleotide in vertebrate genomes under a mutation model**. *Proc Natl Acad Sci U S A* 1990, **87**(12):4692-4696.

4. Takai D, Jones PA: **Comprehensive analysis of CpG islands in human chromosomes 21 and 22**. *Proc Natl Acad Sci U S A* 2002, **99**(6):3740-3745.
