# Supplementary material for: The tendency to recreate ancestral CG dinucleotides in the human genome
Source: BMC Evol Biol. 2011 Jan 5;11:3. doi: 10.1186/1471-2148-11-3 (PMC3025853; doi:10.1186/1471-2148-11-3)
Supplement: Additional file 3 — Number of SNPs and derived-allele frequency for different mutation types in different annotation categories in CEU. [file 1471-2148-11-3-S3.DOC]

|  | Non-CpG island region | | | | CpG island region | | | |
| --- | --- | --- | --- | --- | --- | --- | --- | --- |
|  | Intergenic | | Genic | | Intergenic | | Genic | |
| Mutation type | SNP number | DAF | SNP number | DAF | SNP number | DAF | SNP number | DAF |
| General | 82947 | 0.382 | 74340 | 0.376 | 1060 | 0.42 | 2971 | 0.391 |
| Tsd | 3566 | 0.376 | 4407 | 0.365 | 175 | 0.393 | 460 | 0.379 |
| C-Tsd | 21032 | 0.368 | 18415 | 0.364 | 286 | 0.392 | 785 | 0.366 |
| Tsg | 9605 | 0.403 | 8735 | 0.39 | 113 | 0.468 | 236 | 0.461 |
| C-Tsg | 20450 | 0.389 | 18640 | 0.388 | 139 | 0.478 | 378 | 0.409 |
| Tvd | 382 | 0.39 | 381 | 0.395 | 66 | 0.424 | 198 | 0.383 |
| C-Tvd | 13123 | 0.377 | 10944 | 0.369 | 139 | 0.407 | 447 | 0.39 |
| Tvg | 3208 | 0.371 | 3086 | 0.368 | 55 | 0.442 | 167 | 0.386 |
| C-Tvg | 11595 | 0.388 | 10183 | 0.376 | 100 | 0.405 | 327 | 0.374 |
