# Supplementary material for: The tendency to recreate ancestral CG dinucleotides in the human genome
Source: BMC Evol Biol. 2011 Jan 5;11:3. doi: 10.1186/1471-2148-11-3 (PMC3025853; doi:10.1186/1471-2148-11-3)
Supplement: Additional file 4 — Number of SNPs and derived-allele frequency for different mutation types in different annotation categories in ASN. [file 1471-2148-11-3-S4.DOC]

|  | Non-CpG island region | | | | CpG island region | | | |
| --- | --- | --- | --- | --- | --- | --- | --- | --- |
|  | Intergenic | | Genic | | Intergenic | | Genic | |
| Mutation type | SNP number | DAF | SNP number | DAF | SNP number | DAF | SNP number | DAF |
| General | 88148 | 0.393 | 81244 | 0.389 | 479 | 0.401 | 1143 | 0.376 |
| Tsd | 3761 | 0.381 | 4982 | 0.369 | 65 | 0.346 | 180 | 0.392 |
| C-Tsd | 22247 | 0.38 | 20229 | 0.378 | 117 | 0.408 | 316 | 0.33 |
| Tsg | 10060 | 0.415 | 9493 | 0.407 | 53 | 0.45 | 101 | 0.434 |
| C-Tsg | 21704 | 0.401 | 20190 | 0.399 | 64 | 0.447 | 163 | 0.426 |
| Tvd | 427 | 0.409 | 452 | 0.389 | 30 | 0.462 | 78 | 0.377 |
| C-Tvd | 14076 | 0.388 | 11855 | 0.384 | 69 | 0.319 | 166 | 0.348 |
| Tvg | 3388 | 0.391 | 3421 | 0.383 | 31 | 0.504 | 59 | 0.42 |
| C-Tvg | 12471 | 0.396 | 11127 | 0.39 | 50 | 0.374 | 106 | 0.362 |
