# Supplementary material for: The tendency to recreate ancestral CG dinucleotides in the human genome
Source: BMC Evol Biol. 2011 Jan 5;11:3. doi: 10.1186/1471-2148-11-3 (PMC3025853; doi:10.1186/1471-2148-11-3)
Supplement: Additional file 7 — Number of SNPs and derived-allele frequency for different mutation types in the 9.4 Mb non-genic region. [file 1471-2148-11-3-S7.DOC]

|  | One Outgroup | | Three Outgroups | | After SFS correction | |
| --- | --- | --- | --- | --- | --- | --- |
| Mutation | Number | DAF | Number | DAF | Number | DAF |
| Mutation away from CG | | | | | | |
| CG→TG/CA | 199 | 0.281 | 108 | 0.286 | 205.03 | 0.282 |
| C/G→T/A^1^ | 287 | 0.306 | 228 | 0.296 | 286.85 | 0.305 |
| CG→CC/GG | 5 | 0.198 | 4 | 0.077 | 5.00 | 0.198 |
| C/G→G/C^1^ | 109 | 0.358 | 97 | 0.342 | 109.00 | 0.358 |
| CG→CT/AG | 14 | 0.329 | 5 | 0.458 | 14.02 | 0.330 |
| C/G→A/T^1^ | 97 | 0.247 | 78 | 0.252 | 97.01 | 0.247 |
| Mutation towards CG | | | | | | |
| TG/CA→CG | 226 | 0.406 | 185 | 0.378 | 219.97 | 0.398 |
| T/A→C/G^1^ | 414 | 0.311 | 335 | 0.309 | 414.35 | 0.311 |
| CC/GG→CG | 25 | 0.346 | 24 | 0.334 | 25.00 | 0.346 |
| G/C→C/G^1^ | 114 | 0.351 | 101 | 0.331 | 114.00 | 0.351 |
| CT/AG→CG | 36 | 0.304 | 25 | 0.279 | 35.98 | 0.305 |
| A/T→C/G^1^ | 104 | 0.370 | 84 | 0.354 | 103.99 | 0.370 |

^1.^C/G not preceded by C or followed by G
