# Supplementary material for: The tendency to recreate ancestral CG dinucleotides in the human genome
Source: BMC Evol Biol. 2011 Jan 5;11:3. doi: 10.1186/1471-2148-11-3 (PMC3025853; doi:10.1186/1471-2148-11-3)
Supplement: Additional file 9 — Phylogenetic tree for reliable back-mutations from CA to CG. [file 1471-2148-11-3-S9.DOC]

Rhesus

CG

Gibbon

CG

Orangutan

CA

Gorilla

CA

Chimp

CA

Human2

CA

Human1

CG
